# Difference in expression between AQP1 and AQP5 in porcine endometrium and myometrium in response to steroid hormones, oxytocin, arachidonic acid, forskolin and cAMP during the mid-luteal phase of the estrous cycle and luteolysis

**DOI:** 10.1186/s12958-015-0128-7

**Published:** 2015-12-01

**Authors:** Agnieszka Skowronska, Patrycja Mlotkowska, Soren Nielsen, Mariusz T. Skowronski

**Affiliations:** Department of Human Physiology, Faculty of Medical Sciences, University of Warmia and Mazury in Olsztyn, Warszawska 30, 10-082 Olsztyn, Poland; Department of Animal Physiology, Faculty of Biology and Biotechnology, University of Warmia and Mazury in Olsztyn, Olsztyn, Poland; Department of Health Science and Technology, Faculty of Medicine, Aalborg University, Aalborg, Denmark

**Keywords:** Aquaporins, Endometrium, Myometrium, Estrous cycle, Pig

## Abstract

**Background:**

Recently, we demonstrated *in vitro* that AQP1 and AQP5 in the porcine uterus are regulated by steroid hormones (P4, E2), arachidonic acid (AA), forskolin (FSK) and cAMP during the estrous cycle. However, the potential of the porcine separated uterine tissues, the endometrium and myometrium, to express these AQPs remains unknown. Thus, in this study, the responses of AQP1 and AQP5 to P4, E2 oxytocin (OT), AA, FSK and cAMP in the porcine endometrium and myometrium were examined during the mid-luteal phase of the estrous cycle and luteolysis.

**Methods:**

Real-time PCR and western blot analysis.

**Results:**

Progesterone up-regulated the expression of AQP1/AQP5 mRNAs and proteins in the endometrium and myometrium, especially during luteolysis. Similarly, E2 also stimulated the expression of both AQPs, but only in the endometrium. AA led to the upregulation of AQP1/AQP5 in the endometrium during luteolysis. In turn, OT increased the expression of AQP1/AQP5 mRNAs and proteins in the myometrium during mid-luteal phase. Moreover, a stimulatory effect of forskolin and cAMP on the expression of AQP1/AQP5 mRNAs and proteins in the endometrium and myometrium dominated during luteolysis, but during the mid-luteal phase their influence on the expression of these AQPs was differentiated depending on the type of tissue and the incubation duration.

**Conclusions:**

These results seem to indicate that uterine tissues; endometrium and myometrium, exhibit their own AQP expression profiles in response to examined factors. Moreover, the responses of AQP1/AQP5 at mRNA and protein levels to the studied factors in the endometrium and myometrium are more pronounced during luteolysis. This suggests that the above effects of the studied factors are connected with morphological and physiological changes taking place in the pig uterus during the estrous cycle.

## Background

The uterine wall is composed of two functional compartments, the endometrium and myometrium, surrounded by the perimetrium [1]. The porcine endometrium comprises the luminal epithelial, glandular epithelial and stromal cells [2]. In turn, the porcine myometrium consists of outer and inner layers of smooth muscles; longitudinally- and circularly-oriented to the uterine lumen, respectively [3]. On the basis of *in vitro* studies, Franczak and Kotwica [4], and Wojciechowicz et al. [5] reported that both porcine endometrium and myometrium are steroidogenic tissues producing progesterone, estrogens and androgens. Other reports indicate that porcine endometrium [6–8] as well as myometrium [9, 10] also produce PGE2 and PGF2_alpha_. As a result of ovarian steroid action, the uterine glands expand and the secretory activity increases, becoming the highest at the end of the secretory phase, while during luteolysis, under the influence of oxytocin (OT), uterine fluids and unnecessary cell debris are excreted [11, 12].

Aquaporins (AQPs) are ubiquitous membrane proteins which provide a molecular basis for transmembrane water transport [13]. AQPs are constitutively expressed in the cell membranes, to where they may be trafficked from intracellular vesicles upon appropriate stimulation [14]. So far, at least nine AQP isoforms (including AQP1 and AQP5) have been confirmed in the female reproductive system of humans, rats, mice and pigs [15]. AQP1 is found in many secretory and osmotically-active tissues [16], and is expressed in vascular endothelial cells throughout the body [17, 18]. AQP5 is mainly located in the apical plasma membranes of various secretory glands [19]. Studies with animal models and humans have shown that sufficient expression and proper subcellular targeting of AQP5 channels are necessary to support physiological functions [20–22]. The transport and homeostasis of water in the endometrium is crucial for maintaining proper reproductive processes. Previous reports have demonstrated that the vasculature and epithelium of the uterus have high expression of AQPs [23–25] and that uterine fluid homeostasis is effectively regulated by steroid hormones [26].

Our previous research indicated that AQP1, 5 and 9 are expressed in the porcine uterus, [27, 28], oviduct [29], ovary [30] and peri-ovarian vascular complex [31]. We also observed that these AQPs are differently localized and expressed in these structures during the estrous cycle and early pregnancy. Very recently, we described the response of AQPs (AQP1 and AQP5) to treatments with steroids, OT, arachidonic acid (AA), forskolin (FSK) and cAMP in the uterine explants from cyclic gilts during the mid-luteal phase (Days 10–12) and luteolysis (Days 14–16) [32]. However, to date, the potential of the porcine uterine tissues, endometrium and myometrium to express AQPs has not been studied separately. Therefore, the objectives of the study were: 1/ to describe endometrial and myometrial basal expression of AQP1/AQP5 mRNAs and proteins during Days 10–12 (the mid-luteal phase) and Days 14–16 (luteolysis) of the estrous cycle; 2/ to evaluate whether the steroid hormones (P4 and E2), OT, AA (substrate for prostaglandin synthesis), FSK (adenylate cyclase activator) and cAMP (cyclic adenosine monophosphate; second messenger) may regulate the endometrial and myometrial AQP1 and AQP5 expression at the mRNA and protein levels during these periods. It is assumed that the obtained results will provide useful information on the regulatory mechanism concerning the examined aquaporins in the porcine endometrium and myometrium.

## Methods

### Animals and sample collection

All experiments were performed in accordance with the Animal Ethics Committee, University of Warmia and Mazury in Olsztyn, Poland (AEC approval No. 66/2010/DTN). Tissue samples were recovered from mature cross-bred gilts (Large White × Polish Landrace) on Days 10–12 (*n* = 5) and Days 14–16 (*n* = 5) of the estrous cycle (the stage of luteolysis). Gilts were observed daily for estrous behavior and they were used in the study during their third consecutive normal estrous cycle. The animals were slaughtered at a local abattoir on Days 10–12 (the period of fully-active corpora lutea) and Days 14–16 (the luteolysis period) of the estrous cycle. Additionally, the stage of the cycle was verified by utero-ovarian morphology [33]. Immediately after the slaughter, uteri were placed in ice-cold phosphate-buffered saline (PBS) supplemented with 100 IU/ml penicillin (Polfa, Poland) and 100 μg/ml streptomycin (Polfa, Poland), and transported to the laboratory on ice within ~1 h for *in vitro* tissue culture.

### Preparation of endometrial and myometrial slices

Uterine horns were cut longitudinally and the endometrium and the perimetrium were then separated from the myometrium by careful scraping using scissors. The separation precision was histologically verified under a dissecting microscope. Tissues were cut into small pieces and washed twice with sterile PBS. Individual endometrial and myometrial slices (200–210 mg weight, 3 mm thick) were placed separately in culture vials containing 2 ml Medium 199 supplemented with 0.1 % BSA (Sigma), 20 μg nystatin (Sigma) and 20 μg gentamicin (Krka, Novo Mesto, Slovenia) and then pre-incubated under an atmosphere of 95 % O_2_ and 5 % CO_2_ at 37 °C for 18 h. After preincubation, the culture medium was replaced with fresh medium and the slices were incubated with vehicle (control) or P4 (10^−5^ M; Sigma), E2 (10^−9^ M; Sigma), OT (10^−7^ M; Sigma), AA (10^−5^ M; Sigma), FSK (10 μg/mL; Sigma) and cpt-cAMP analogue (200 μM; Sigma) for 3 or 24 h. All treatments were performed in triplicate.

### Total RNA isolation, cDNA synthesis and real-time polymerase chain reaction analysis

Total RNA was extracted from the uterine explants collected after in vitro culture (*n* = 5 cultures), using fenozol (A&A Biotechnology, Gdansk Poland) in accordance with the manufacturer’s instructions. RNA quality and quantity were determined with spectrophotometry (NanoDrop ND-1000, Thermo Scientific, Wilmington, DE, USA). Total RNA samples were transcribed to cDNA using an Enhanced Avian HS RT-PCR Kit (Sigma) and a mix of dNTPs and random hexamers as primers. Real-Time PCR was performed in duplicate for each sample using a 7300 Real-Time PCR system and SYBR®Green PCR Master Mix (Life Technologies, Grand Island, NY, USA) and specific primers for *AQP1* and *AQP5* as described previously [32]. Samples for the specificity control, non-template controls and dissociation curve analysis of the amplified products were used for each reaction. The specificity of amplifications was further validated with electrophoresis of the PCR products in a 2 % agarose gel and the obtained amplicons, after extraction from the gel, were then submitted to automated sequencing using a 3730 xl DNA Analyzer (Life Technologies). The levels of gene expression were calculated with the ΔΔ Ct method and normalized using the geometrical means of two reference gene expressions, glyceraldehyde 3-phosphate dehydrogenase (*GAPDH*) and *18S rRNA*.

### SDS-PAGE and Western blot

Following isolation, the tissues were immediately placed in ice-cold dissection buffer (0.3 M sucrose, 25 mM imidazol, 1 mM EDTA in ddH_2_O, pH 7.2) containing 8.4 μM leupeptin and 0.4 mM pefabloc. As described previously [32], the membranes were incubated overnight at 5 °C with anti-AQPs or beta-actin antibodies, then with horseradish peroxidase-conjugated secondary antibody (P448, diluted 1:3,000, Dako A/S, Glostrup, Denmark) in PBS-T for 1 h. After washing with PBS-T, the antibody-antigen reaction sites were visualized with an enhanced chemiluminescence (ECL) system (Amersham Pharmacia Biotech, Little Chalfont, UK) and exposed to photographic film (Hyperfilm ECL, RPN3103K, Amersham Pharmacia Biotech, Little Chalfont, UK). The results of Western blotting were quantified by densitometric scanning of immunoblots with GelScan for Windows ver. 1.45 software (Kucharczyk, Poland). The data was expressed as the AQP protein: actin protein ratio in OD units. In our previous study, we demonstrated that pre-incubation of anti-AQP1 and anti-AQP5 antibodies with the respective AQP antigens (immunizing peptides) prevented labeling in the porcine uterine tissues [28].

### Statistical analysis

All numerical data were analyzed by one-way ANOVA and a least significant difference (LSD) *post hoc* test and were reported as the means ± S.E.M. from five independent observations. Statistical analyses were performed using Statistica software (Stat Soft Inc., Tulsa, USA). Values for *p* <0.05 were considered statistically significant.

## Results

### AQP1 mRNA expression in porcine endometrial and myometrial tissues

**Fig. 1:**
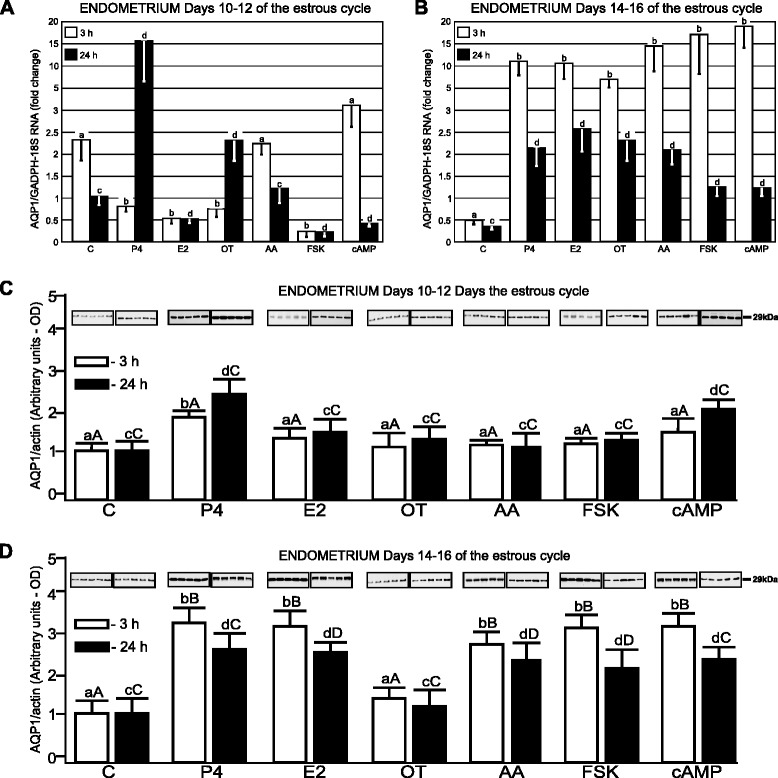
AQP1 mRNA and protein expression in porcine endometrial explants. The expression of *AQP1* mRNA in the endometrial tissue harvested from pigs (*n* = 5) on Days 10–12 (**a**) and 14–16 (**b**) of the estrous cycle after treatment with steroids (P4, E2), oxytocin (OT), arachodonic acid (AA), forskolin (FSK) and cAMP for 3 and 24 h. The expression of the studied gene, determined by real-time PCR, is presented as means ± SEM in relation to the expression of reference genes, *GAPDH* and *18 sRNA*. Different letters indicate significant differences (*p* <0.05) between each treatment and respective control values for 3- (**a**, **b**) or 24-h (**c**, **d**) incubations. The protein content of AQP1 was determined by semi-quantitative Western blot analysis, in the endometrial explants harvested from pigs (*n* = 5) on Days 10–12 (**c**) and 14–16 (**d**) of the cycle after treatment with the above-mentioned factors for 3 and 24 h. The data is presented as means ± SEM in relation to β-actin tissue content. Different letters indicate significant differences (*p* <0.05) between each treatment and respective control values for 3- (**a**, **b**) or 24-h (**c**, **d**) incubations. Different large letters indicate significant differences (*p* <0.05) between the same treatments for 3- (**a**, **b**) or 24-h (**c**, **d**) incubations for different periods (Days 10–12 and 14–16) of the estrous cycle

The control abundance of *AQP1* transcript from the mid-luteal phase (Days 10–12) harvested from the endometrial tissue was about 3.5-fold higher than from luteolysis (Days 14–16) during 3-h incubation (*p* <0.05; Fig. 1a and b). The endometrial expression of *AQP1* mRNA on Days 10–12 decreased after a 3-h treatment with P4 and OT (*p* <0.05), but during longer incubation (24 h), these treatments significantly (*p* <0.05) increased it (Fig. 1a). Treatments with E2 and FSK decreased (*p* <0.05) *AQP1* mRNA expression in the endometrium from the mid-luteal phase, after 3- and 24-h incubations. During luteolysis, the expression of *AQP1* was strongly elevated in response to P4, E2, OT, AA, FSK and cAMP (*p* <0.05) in the endometrium after 3- and 24-h (Fig. 1b).

**Fig. 2:**
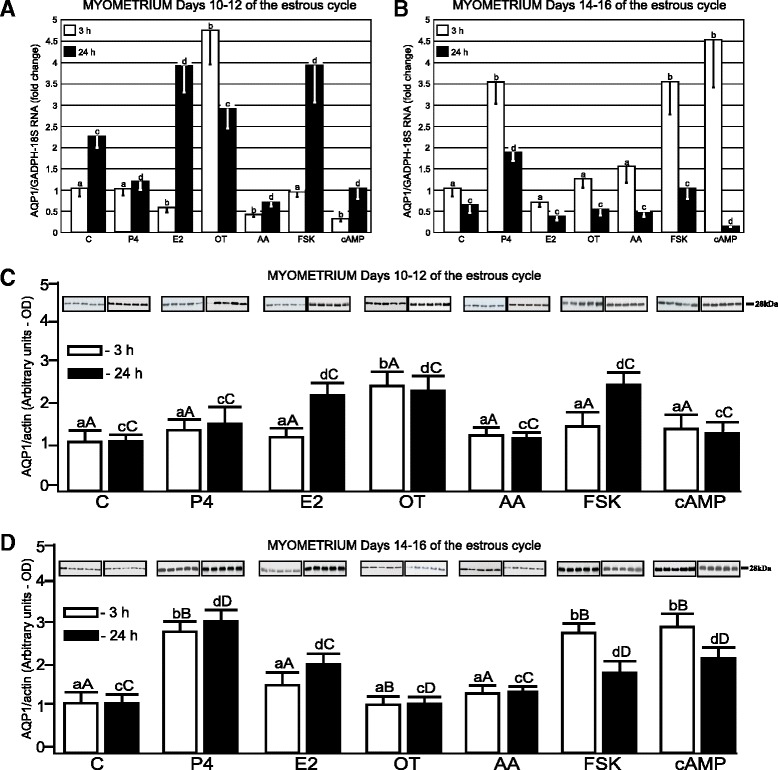
AQP1 mRNA and protein expression in porcine myometrial explants. The expression of *AQP1* mRNA in the myometrial tissue harvested from pigs (*n* = 5) on Days 10–12 (**a**) and 14–16 (**b**) of the estrous cycle after treatment with steroids (P4, E2), oxytocin (OT), arachodonic acid (AA), forskolin (FSK) and cAMP for 3 and 24 h. The expression of the studied gene, determined by real-time PCR, is presented as means ± SEM in relation to the expression of reference genes, *GAPDH* and *18 sRNA*. Different letters indicate significant differences (*p* <0.05) between each treatment and respective control values for 3- (**a**, **b**) or 24-h (**c**, **d**) incubations. The protein content of AQP1 was determined by semi-quantitative Western blot analysis, in the myometrial explants harvested from pigs (*n* = 5) on Days 10–12 (**c**) and 14–16 (**d**) of the cycle after treatment with the abovementioned factors for 3 and 24 h. The data is presented as means ± SEM in relation to β-actin tissue content. Different letters indicate significant differences (*p* <0.05) between each treatment and respective control values for 3- (**a**, **b**) or 24-h (**c**, **d**) incubations. Different large letters indicate significant differences (*p* <0.05) between the same treatments for 3- (**a**, **b**) or 24-h (**c**, **d**) incubations for different periods (Days 10–12 and 14–16) of the estrous cycle

In the myometrium (Fig. 2a), P4 decreased (*p* <0.05) *AQP*1 expression after 24-h incubation on Days 10–12. Treatment of this tissue with E2 for 3-h also significantly decreased (*p* <0.05) *AQP1* mRNA expression, but elevated (*p* <0.05) it after 24-h incubation. Incubation of the tissue with OT elevated *AQP1* mRNA expression, but with AA and cAMP it decreased after 3-h incubation (*p* <0.05). In turn, during longer incubation (24 h), FSK elevated AQP1 gene expression (*p* <0.05). During luteolysis, a stimulation (*p* <0.05) of *AQP1* expression in the myometrial tissue was observed after treatments with P4 (after 3 and 24 h), FSK and cAMP (after 3-h; *p* <0.05). Incubation with E2, OT and AA did not affect the expression of *AQP1* mRNA in this tissue (Fig. 2b).

### AQP5 mRNA expression in porcine endometrial and myometrial tissues

**Fig. 3:**
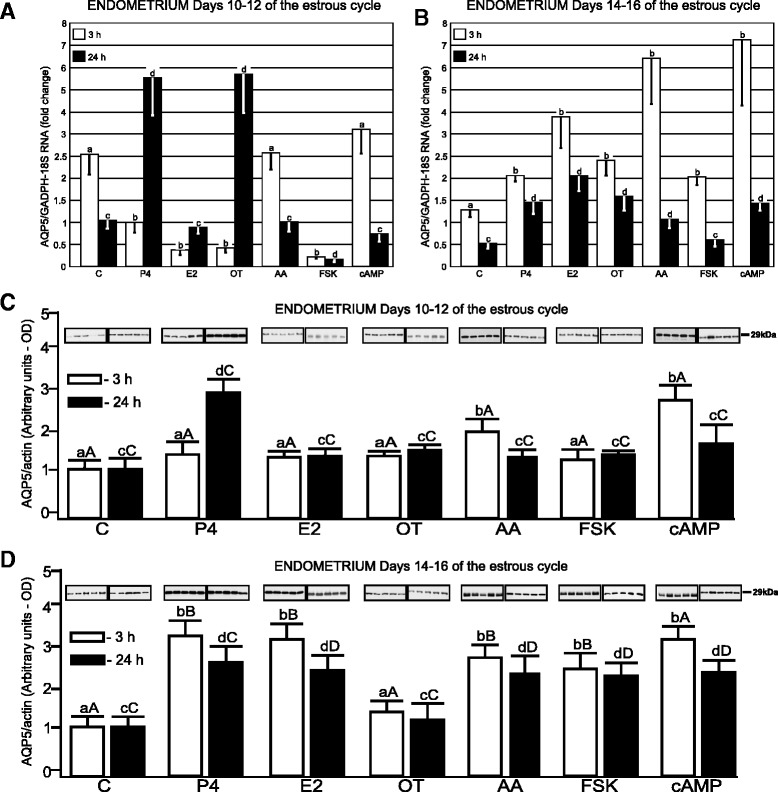
AQP5 mRNA and protein expression in porcine endometrial explants. The expression of *AQP5* mRNA in the endometrial tissue harvested from pigs (*n* = 5) on Days 10–12 (**a**) and 14–16 (**b**) of the estrous cycle after treatment with steroids (P4, E2), oxytocin (OT), arachodonic acid (AA), forskolin (FSK) and cAMP for 3 and 24 h. The expression of the studied gene, determined by real-time PCR, is presented as means ± SEM in relation to the expression of reference genes, *GAPDH* and *18 sRNA*. Different letters indicate significant differences (*p* <0.05) between each treatment and respective control values for 3- (**a**, **b**) or 24-h (**c**, **d**) incubations. The protein content of AQP5 was determined by semi-quantitative Western blot analysis, in the endometrial explants harvested from pigs (*n* = 5) on Days 10–12 (**c**) and 14–16 (**d**) of the cycle after treatment with the abovementioned factors for 3 and 24 h. The data is presented as means ± SEM in relation to β-actin tissue content. Different letters indicate significant differences (*p* <0.05) between each treatment and respective control values for 3- (**a**, **b**) or 24-h (**c**, **d**) incubations. Different large letters indicate significant differences (*p* <0.05) between the same treatments for 3- (**a**, **b**) or 24-h (**c**, **d**) incubations for different periods (Days 10–12 and 14–16) of the estrous cycle

**Fig. 4:**
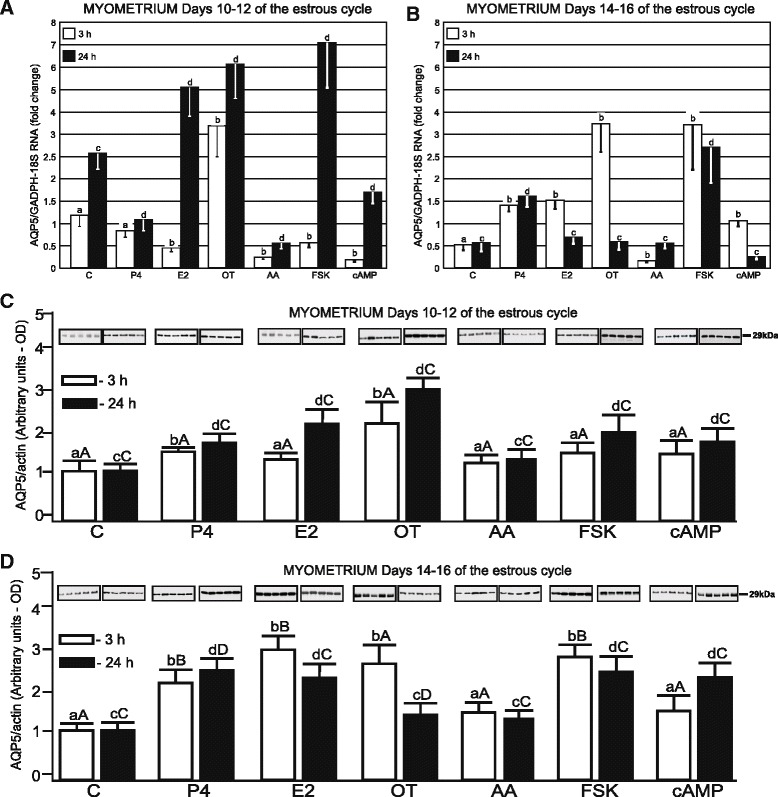
AQP5 mRNA and protein expression in porcine myometrial explants. The expression of *AQP5* mRNA in the myometrial tissue harvested from pigs (*n* = 5) on Days 10–12 (**a**) and 14–16 (**b**) of the estrous cycle after treatment with steroids (P4, E2), oxytocin (OT), arachodonic acid (AA), forskolin (FSK) and cAMP for 3 and 24 h. The expression of the studied gene, determined by real-time PCR, is presented as means ± SEM in relation to the expression of reference genes, *GAPDH* and *18 sRNA*. Different letters indicate significant differences (*p* <0.05) between each treatment and respective control values for 3- (**a**, **b**) or 24-h (**c**, **d**) incubations. The protein content of AQP5 was determined by semi-quantitative Western blot analysis, in the myometrial explants harvested from pigs (*n* = 5) on Days 10–12 (**c**) and 14–16 (**d**) of the cycle after treatment with the abovementioned factors for 3 and 24 h. The data is presented as means ± SEM in relation to β-actin tissue content. Different letters indicate significant differences (*p* <0.05) between each treatment and respective control values for 3- (**a**, **b**) or 24-h (**c**, **d**) incubations. Different large letters indicate significant differences (*p* <0.05) between the same treatments for 3- (**a**, **b**) or 24-h (**c**, **d**) incubations for different periods (Days 10–12 and 14–16) of the estrous cycle

The control expression of *AQP5* transcript in the endometrial slices on Days 10–12 of the estrous cycle was 2.5-fold and 2.0-fold higher than on Days 14–16, after 3- and 24-h incubation, respectively (*p* <0.05; Fig. 3a and b). Treatment of this tissue with E2, OT and FSK after 3 h decreased AQP5 gene expression (*p* <0.05). Treatments with P4 and OT significantly stimulated *AQP5* gene expression in this tissue following longer incubation (*p* <0.05). In turn, treatments with E2 (3-h) and FSK (3- and 24-h) significantly decreased (*p* <0.05) *AQP5* gene expression in endometrial tissues on Days 10–12 of the estrous cycle. During luteolysis (Days 14–16), all treatments (P4, E2, OT, AA, FSK and cAMP) stimulated (*p* <0.05) the expression of *AQP5* for 3 h (Fig. 3b). The expression of *AQP5* mRNA in the myometrium from Days 10–12 was significantly decreased after 24-h treatment with P4 (*p* <0.05, Fig. 4a).

The control expression of *AQP5* transcript in the myometrial slices during the mid-luteal phase (Days 10–12) was significantly higher than during luteolysis (Days 14–16) after both short (3 h) and long (24 h) incubations. On Days 10–12 of the cycle, the myometrial *AQP5* expression was reduced by E2, AA, FSK and cAMP following short incubation (Fig. 4a) as well by P4, AA and cAMP during longer incubation. In contrast, treatments with OT for 3 h as well as with E2, OT, FSK for 24 h stimulated *AQP5* mRNA expression during this period. During luteolysis (Days14-16), increased *AQP5* gene expression in myometrial explants was observed in response to P4 and FSK (*p* <0.05, Fig. 4b) after both incubation periods. Treatments of the myometrial slices for 3 h with E2, OT and cAMP were significantly (*p* <0.05) increased, but decreased by AA (Fig. 4b).

### Protein content of AQP1 in endometrial and myometrial explants from Days 10–12 and 14–16 of the estrous cycle

AQP1 protein expression in the endometrium from Days 10–12 increased (*p* <0.05) after treatment with P4 during 3- and 24-h incubation (Fig. 1c). However, cAMP stimulated (*p* <0.05) AQP1 expression only after longer incubation (24-h). In turn, treatment with E2, OT, AA and FSK did not influence AQP1 protein expression in the endometrium during the mid-luteal phase. During luteolysis (Days 14–16), AQP1 protein expression in the endometrium significantly increased after treatment with P4, E2, AA, FSK and cAMP during 3- and 24-h incubation (Fig. 1D; *p* <0.05).

AQP1 protein expression in the myometrium was significantly up-regulated (*p* <0.05) by OT (after both incubation periods) during the mid-luteal phase (Fig. 2c). E2 and FSK elevated this AQP content (*p* <0.05) only after 24-h incubation. During luteolysis, AQP1 protein content significantly increased (*p* <0.05) after treatment with P4, FSK and cAMP after 3 and 24 h as well as with E2 only after longer incubation (Fig. 2d).

Comparison of the endometrial AQP1 response to applied factors between studied periods indicated that E2, AA, FSK during 3- and 24-h treatments as well as P4 and cAMP after 3-h incubation more effectively stimulated (*p* <0.05) this AQP expression during luteolysis than the mid-luteal phase (Fig. 1c and d). In turn, during the mid-luteal phase, the myometrial AQP1 expression was more sensitive (*p* <0.05) to stimulatory action of OT during both incubation periods (Fig. 2c and d).

### Protein content of AQP5 in endometrial and myometrial explants from Days 10–12 and 14–16 of the estrous cycle

The expression of AQP5 protein in endometrial explants, representing the mid-luteal phase increased (*p* <0.05) after longer treatment with P4 and, in response to AA and cAMP, only after shorter incubation (Fig. 3c and d). During luteolysis, the endometrial AQP5 protein content was significantly (*p* <0.05) higher after treatment with P4, E2, AA, FSK and cAMP. In addition, the endometrial AQP5 protein expression appeared to be more sensitive to treatment with E2, AA, FSK and cAMP (during both incubation periods) as well as P4 (short incubation) during luteolysis than during the mid-luteal phase.

The expression of AQP5 in myometrial explants during the mid-luteal phase significantly increased (*p* <0.05) after treatment with P4 and OT during both incubation periods as well as in response to E2, FSK and cAMP after longer incubation (Fig. 4c). During luteolysis, the myometrial AQP5 protein content was elevated (*p* <0.05) in the presence of P4, E2 and FSK after 3- and 24-h incubations as well as OT after 3-h and cAMP after 24-h treatments (Fig. 4d). Moreover, the responsiveness of myometrial AQP5 to P4 (both incubation periods) and E2, FSK (short incubation) was more pronounced (*p* <0.05) on Days 14–16 than on Days 10–12 of the cycle. Conversely, OT was more effective during the mid-luteal phase than during luteolysis (Fig. 4c and d).

## Discussion

In this study, the responses of AQP1 and AQP5 to P4, E2, OT, AA, FSK and cAMP in the porcine endometrium and myometrium during the mid-luteal phase and luteolysis are delineated. The basal expression of *AQP1* and *AQP5* mRNA in the endometrium on Days 10–12 appeared to be higher (approx. 5- and 2-fold, respectively) than on Days 14–16 of the estrous cycle. In the myometrium, the *AQP5* transcript was also higher on Days 10–12 versus Days 14–16 of the cycle. In turn, the responses of AQPs mRNAs and their proteins to the examined factors in the myometrium and endometrium were more pronounced during luteolysis than the mid-luteal phase.

In previous studies performed with rodents, the expression of AQP1 and AQP5 appeared to be sensitive to estrogens and/or P4 regulatory action [23, 34–37]. Hildebrand et al. [38] confirmed AQP1 expression in the human endometrium during a normal cycle and found its increase after treatment with mifepristone (an anti-progestogenic steroid). In the present study, P4 unanimously up-regulated AQP1 and AQP5 mRNA and protein expression in the endometrium during luteolysis as well as in the mid-luteal phase during longer incubation, but the responses were differentiated during shorter incubation. In the myometrium, the expression of AQPs mRNAs and proteins were up-regulated in the presence of P4 during luteolysis. In turn, on Days 10–12 of the cycle, only the abundance of both AQPs proteins increased. In our recent in vitro studies [32], performed with the porcine uterine explants, consisting of endometrium and myometrium, P4 evidently stimulated the expression of both AQPs at the protein level, but mostly inhibited it at the transcriptional level. The data suggests that P4 is an important regulator of AQP1 and AQP5 expression in the porcine uterus, particularly in the endometrium. In addition, it should be noted that under physiological conditions, during the mid-luteal phase, P4 secreted by corpus luteum (CL) is to a certain degree responsible for modulation of the uterine expression of AQPs, while during luteolysis, locally-produced P4 (by the endometrium and myometrium [5, 39]) seems to be mainly engaged in this regulation. In the present experiment, E2 did not affect AQP1 and AQP5 gene and protein expression in the endometrial slices from the mid-luteal phase. In contrast, during luteolysis, E2 evidently exerted stimulatory effects on the endometrial expression of both AQPs at mRNA and protein level. The treatment of myometrial tissue with E2 (for 24 h) similarly stimulated these AQPs during the mid-luteal phase, but during luteolysis only their proteins were up-regulated. In turn, in the experiment performed with whole uterine tissue explants [32], E2 clearly stimulated the expression of AQP1 and AQP5 at the protein level during both studied periods, but its influence on the expression of *AQPs* mRNA was differentiated. In literature, data concerning the effect of steroid hormones on the in vitro expression of AQP1/5 in separated uterine tissues from cyclic females is not available. Li et al. [35] observed the effect of E2 on the expression of water channel gene (AQP-CHIP). It was shown that endometrial AQP2 expression correlates with serum E2 and P4 concentrations [40] and that, in the rat uterus, the *AQP5* gene is activated by estrogens [36] and AQP5 protein is up-regulated by P4 [37]. In turn, AQP1 appeared to be slightly regulated by E_2_ in the myometrium [23]. On the basis of our results, it seems that endometrium and myometrium uterine tissues exhibit their own profiles of AQPs expression in response to P4 and E2. Moreover, it appears that the action of steroid hormones within the uterus during the estrous cycle also includes their influence on the expression of AQPs, which reacted with quantitative and qualitative changes related to different processes, such as increasing secretory activity and promoting angiogenesis and edema [28, 41].

Previous studies have suggested that OT might be implicated in the regulation of AQP expression in the uterus [32, 42]. It has been reported that OT receptors are present in the endometrium and myometrium of cyclic gilts [43, 44]. In the present study, OT increased the expression of AQP1/AQP5 mRNAs and proteins in the myometrial slices during the mid-luteal phase, but only AQP5 mRNA and protein (3 h) during luteolysis. In endometrial tissue, some responses in the expression of AQPs transcript were noted, without any changes at the protein level. The discrepancy between the reaction of whole and separated uterine tissues to treatment with OT is intriguing. It might result from the fact that conditions in separated tissues do not entirely reflect the situation in the whole tissue explant [32], which encompasses the endometrial/myometrial interactions based on paracrine effects. It is known that communication between the epithelium, stroma and myometrium is very complex and, among others, involves locally-produced steroid hormones as well as growth factors and cytokines [39], which may affect uterine expression of AQPs. Moreover, studies performed with rats also indicate the involvement of OT in the regulation of AQP5, especially during late pregnancy [42]. Collectively, it might be stated that OT is connected with the uterine expression of AQPs in pigs, but full explanation of its role in this process requires further study.

Arachidonic acid is important for biosynthesis of prostaglandins, which play an essential role in the regulation of reproductive processes, including luteolysis and maternal recognition of pregnancy [45–47]. In the present study, AA did lead to up-regulation of AQP1/AQP5 mRNAs and proteins in the endometrial slices during luteolysis. This is in agreement with our recent studies, which demonstrated that the expression of AQP proteins is stimulated in the explants of the entire uterus during this period [32]. The observation corresponds well with the physiologically-increased release of PGF2_alpha_ from the endometrium during luteolysis to cause CL regression [48]. In turn, the endometrial synthesis of PGs is low in the mid-luteal phase of the cycle [48, 49]. Furthermore, we did not observe any response of AQP1 and AQP5 proteins to AA in the myometrium during the studied periods. Importantly, our current findings and recently published data [32] suggest that PGF2_alpha_ may affect the endometrial expression of AQPs during luteolysis. However, further studies are required to verify this hypothesis.

Our previous studies demonstrated the expression of AQP1 and AQP5 in the porcine uterine explants in response to forskolin and cAMP [32]. In the present study, the stimulatory effects of forskolin and cAMP on the expression of AQP1/AQP5 mRNAs and proteins in the endometrium and myometrium clearly dominated during luteolysis. In contrast, results concerning the expression of AQPs during the mid-luteal phase of the cycle were differentiated, e.g., in the endometrium there was no response or inhibition at the mRNA level, but no response or stimulation at the protein level. Other studies have confirmed the involvement of cAMP in the regulation of AQP5 gene expression in murine lung epithelial cell [50] and human WISH cell [51] lines as well as human fetal membranes [52] and amnion epithelia [53]. Our data indicate the participation of the adenylate cyclase/cAMP pathway in the intracellular transduction of signals connected with the regulation of AQP1 and AQP5 expression in the porcine uterine tissues, which seems to be augmented during luteolysis, but differentiated during the mid-luteal phase, depending on the type of tissue and the duration of incubation.

## Conclusions

In summary, the present study conducted with separated uterine tissues has confirmed the participation of steroid hormones (E2 and P4), OT and AA in the regulation of AQP1 and AQP5 expression at the levels of mRNA and protein in the endometrium and myometrium of cyclic gilts during the mid-luteal phase and luteolysis. The responses of both AQPs to the examined factors exhibited fluctuations, depending on the type of tissue as well as the stage of the estrous cycle and the incubation time. Moreover, the study indicated the involvement of the adenylate cyclase/cAMP pathway in the regulation of AQP1 and AQP5 in the porcine endometrium and myometrium. The above regulations seem to be important for cyclic changes connected with the preparation of an intrauterine environment for proper development of embryos/fetuses and successful reproduction.

## References

[1] Spencer TE, Johnson GA, Burghard RC, Bazer FW (2004). Progesterone and placental hormone actions on the uterus: insights from domestic animals. Biol Reprod.

[2] Blackwell DM, Speth RC, Mirando MA (2003). Morphometric analysis of the uterine endometrium of swine on days 12 and 16 postestrus. Anat Rec Part A.

[3] Thilander G, Rodrigez-Martinez H (1989). Fine structure of the porcine myometrium during the oestrous cycle. Acta Anat.

[4] Franczak A, Kotwica G (2008). Secretion of estradiol-17beta by porcine endometrium and myometrium during early pregnancy and luteolysis. Theriogenology.

[5] Wojciechowicz B, Kotwica G, Kolakowska J, Franczak A (2013). The activity and localization of 3β-hydroxysteroid dehydrogenase/Δ(5)-Δ(4) isomerase and release of androstenedione and progesterone by uterine tissues during early pregnancy and the estrous cycle in pigs. J Reprod Dev.

[6] Uzumcu M, Carnahan KG, Braileanu GT, Mirando MA (2000). Oxytocin-stimulated phosphoinositide hydrolysis and prostaglandin F2alpha secretion by luminal epithelial, glandular epithelial and stromal cells from pig endometrium. II. Responses of cyclic, pregnant and pseudo-pregnant pigs on days 12 and 16 postoestrus. Reprod Fertil Dev.

[7] Ashworth MD, Ross JW, Hu J, White FJ, Stein DR, Desilva U (2006). Expression of porcine endometrial prostaglandin synthase during the estrous cycle and early pregnancy and following endocrine disruption of pregnancy. Biol Reprod.

[8] Blitek A, Kiewisz J, Waclawik A, Kaczmarek MM, Ziecik AJ (2010). Effect of steroids on HOXA10 mRNA and protein expression and prostaglandin production in the porcine endometrium. J Reprod Dev.

[9] Franczak A, Kotwica G, Kurowicka B, Oponowicz A, Wacławek-Potocka J, Petroff BK (2006). Expression of enzymes of cyclooxygenase pathway and secretion of prostaglandin E2 and F2α by porcine myometrium during luteolysis and early pregnancy. Theriogenology.

[10] Franczak A, Zmijewska A, Kurowicka B, Wojciechowicz B, Kotwica G (2010). Interleukin 1β-induced synthesis and secretion of prostaglandin E2 in the porcine uterus during various periods of pregnancy and the estrous cycle. J Physiol Pharmacol.

[11] Krzymowski T, Stefanczyk-Krzymowska S (2008). The role of the endometrium in endocrine regulation of the animal oestrous cycle. Reprod Domest Anim.

[12] Wasowska B (1999). Relationship between the blood supply, course of energy processes, secretion PGF2α and apoptosis in the porcine endometrium during the estrous cycle. (in Polish).

[13] Agre P, King LS, Yasui M, Guggino WB, Ottersen OP, Fujiyoshi Y (2002). Aquaporin water channels-from atomic structure to clinical medicine. J Physiol.

[14] Liu H, Wintour EM (2005). Aquaporins in development – a review. Reprod Biol Endo.

[15] Zhu C, Jiang Z, Bazer FW, Johnson GA, Burghardt RC, Wu G (2015). Aquaporins in the female reproductive system of mammals. Front Biosci Landmark.

[16] King LS, Kozono D, Agre P (2004). From structure to disease: the evolving tale of aquaporin biology. Nat Rev Mol Cell Biol.

[17] Mobasheri A, Marples D (2004). Expression of the AQP-1 water channel in normal human tissues: a semi-quantitative study using tissue microarray technology. Am J Physiol Cell Physiol.

[18] Bondy C, Chin E, Smith BL, Preston GM, Agre P (1993). Developmental gene expression and tissue distribution of the CHIP28 water-channel protein. Proc Natl Acad Sci U S A.

[19] Nielsen S, King LS, Christensen BM, Agre P (1997). Aquaporins in complex tissue. II. Subcellular distribution in respiratory and glandular tissues of rat. Am J Physiol.

[20] Ma T, Fukuda N, Song Y, Matthay MA, Verkman AS (2000). Lung fluid transport in aquaporin-5 knockout mice. J Clin Invest.

[21] Song Y, Verkman AS (2001). Aquaporin-5 Dependent Fluid Secretion in Airway Submucosal Glands. J Biol Chem.

[22] Tsubota K, Hirai S, King LS, Agre P, Ishida N (2001). Defective cellular trafficking of lacrimal gland aquaporin-5 in Sjögren’s syndrome. Lancet.

[23] Jablonski EM, McConnell NA, Hughes FM, Huet-Hudson YM (2003). Estrogen regulation of aquaporins in the mouse uterus: potential roles in uterine water movement. Biol Reprod.

[24] Richard C, Gao JU, Brown N, Reese J (2003). Aquaporin water channel genes are differentially expressed and regulated by ovarian steroids during the peri-implantation period in the mouse. Endocrinology.

[25] Feng C, Chao-Chao S, Wang T, Rong-Huan H, Jian-Zhong S, He-Feng H (2008). Decreased expression of endometrial vessel AQP1 and endometrial epithelium AQP2 related to an ovulatory uterine bleeding in premenopausal women. Menopause.

[26] Chen Q, Zhang Y, Elad D, Jaffa AJ, Cao Y, Ye X (2013). Navigating the site for embryo implantation: Biomechanical and molecular regulation of intrauterine embryo distribution. Mol Aspects Med.

[27] Skowronski MT, Kwon TH, Nielsen S (2009). Immunolocalization of aquaporin 1, 5, in 9 in the female pig reproductive system. J Histochem Cytochem.

[28] Skowronski MT (2010). Distribution and quantitative changes in amounts of aquaporin 1, 5 and 9 in the pig uterus during the estrous cycle and early pregnancy. Reprod Biol Endocrinol.

[29] Skowronski MT, Skowronska A, Nielsen S (2011). Fluctuation of aquaporin 1, 5, and 9 expression in the pig oviduct during the estrous cycle and early pregnancy. J Histochem Cytochem.

[30] Skowronska A, Mlotkowska P, Eliszewski M, Nielsen S, Skowronski MT (2015). Expression of aquaporin 1, 5 and 9 in the ovarian follicles of cycling and early pregnant pigs. Physiol Res.

[31] Skowronski MT, Frackowiak L, Skowronska A (2011). Expression of aquaporin 1 in the pig peri-ovarian vascular complex during the estrous cycle and early pregnancy. Reprod Biol.

[32] Skowronska A, Młotkowska P, Wojciechowicz B, Okrasa S, Nielsen S, Skowronski MT (2015). Progesterone, estradiol, arachidonic acid, oxytocin, forskolin and cAMP influence on aquaporin 1 and 5 expression in porcine uterine explants during the mid-luteal phase of the estrous cycle and luteolysis: an in vitro study. Reprod Biol Endocrinol.

[33] Akins EL, Morrisete MC (1968). Gross ovarian changes during the estrous cycle of swine. Am J Vet Res.

[34] Lindsay LA, Murphy CR (2004). Aquaporin-1 increases in the rat myometrium during early pregnancy. J Mol Histol.

[35] Li XJ, Yu HM, Koide SS (1997). Regulation of water channel gene (AQP-CHIP) expression by estradiol and anordiol in rat uterus. Yao Xue Xue Bao.

[36] Kobayashi M, Takahashi E, Miyagawa S, Watanabe H, Iguchi T (2006). Chromatin immunoprecipitation-mediated target identification proved aquaporin 5 is regulated directly by estrogen in the uterus. Genes Cells.

[37] Lindsay LA, Murphy CR (2006). Redistribution of aquaporins 1 and 5 in the rat uterus is dependent on progesterone: a study with light and electron microscopy. Reproduction.

[38] Hildenbrand A, Stavreus-Evers A, Lalitkumar PG, Nielsen S, Mints M, Gemzell-Danielsson K (2008). Aquaporin 1 is expressed in the human endometrium during normal cycle and increases after mifepristone treatment. Int J Mol Med.

[39] Okrasa S, Franczak A, Zmijewska A, Wojciechowicz B, Dziekonski M, Martyniak M (2014). The uterine secretory activity and its physiological changes in the pig. Acta Biol Cracoviensia.

[40] He RH, Sheng JZ, Luo Q, Jin F, Wang B, Qian YL (2006). Aquaporin-2 expression in human endometrium correlates with serum ovarian steroid hormones. Life Sci.

[41] Goodger AM, Rogers PA (1995). Blood vessel growth in the endometrium. Microcirculation.

[42] Ducza E, Seres AB, Hajagos-Toth J, Falkay G, Gaspar R (2014). Oxytocin regulates the expression of aquaporin 5 in the late-pregnant rat uterus. Mol Reprod Dev.

[43] Okano A, Okuda K, Takahashi M, Schams D (1996). Oxytocin receptors in porcine endometrium during the estrous cycle and early pregnancy. Animal Reprod Sci.

[44] Franczak A, Ciereszko R, Kotwica G (2005). Oxytocin (OT) action in uterine tissues of cyclic and early pregnant gilts: OT receptors concentration, prostaglandin F(2)alpha secretion, and phosphoinositide hydrolysis. Anim Reprod Sci.

[45] Norman SJ, Poyser N (2000). Effects of inhibitors of arachidonic acid turnover on the production of prostaglandins by the guinea-pig uterus. J Reprod Fertil.

[46] Hertelendy F, Zakar T (2004). Prostaglandins and the myometrium and cervix. Prostaglandin Leukotriens Essential Fatty Acids.

[47] Kennedy TG, Gillio-Meina C, Phang SH (2007). Prostaglandins and the initiation of blastocyst implantation and decidualization. Reproduction.

[48] Gleeson AR, Thorburn GD, Cox RI (1974). Prostaglandin F concentrations in theutero-ovarian venous plasma of the sow during the late luteal phase of the oestrous cycle. Prostaglandins.

[49] Moeljono MPE, Thatcher WW, Bazer FW, Frank M, Owens LJ, Wilcox CJ (1977). A study of prostaglandin F2 as the luteolysin in swine. II. Characterization and comparison of prostaglandin E estrogens and progestin concentrations in utero-ovarian vein plasma of non-pregnant and pregnant gilts. Prostaglandins.

[50] Yang F, Kawedia JD, Menon AG (2003). Cyclic AMP regulates aquaporin 5 expression at both transcriptional and post-transcriptional levels through a protein kinase A pathway. J Biol Chem.

[51] Wang S, Chen J, Au KT, Ross MG (2003). Expression of aquaporin 8 and its up-regulation by cyclic adenosine monophosphate in human WISH cells. Am J Obstet Gynecol.

[52] Wang S, Amidi F, Beall M, Gui L, Ross MG (2006). Aquaporin 3 expression in human fetal membranes and its up-regulation by cyclic adenosine monophosphate in amnion epithelial cell culture. J Soc Gynecol Investig.

[53] Wang S, Amidi F, Yin S, Beall M, Ross MG (2007). Cyclic adenosine monophosphate regulation of aquaporin gene expression in human amnion epithelia. Reprod Sci.

